# An electrochemical hypothesis of earthquakes exploring a theoretical link between radiated seismic energy and Pourbaix potential

**DOI:** 10.1038/s41598-026-40629-w

**Published:** 2026-02-17

**Authors:** Atanu Das, Sankar Prasad Bag

**Affiliations:** 1 Manipal Institute of Technology, Manipal Academy of Higher Education, Manipal, 576104 India; 2 School of Electronics and Communication Engineering, REVA University, Bangalore, 560064 India

**Keywords:** Seismology, Geophysics

## Abstract

Earthquakes are measured using well-defined seismic parameters such as seismic moment ($M_o$), moment magnitude ($M_w$), and released elastic energy (E). However, the mechanism by which this tremendous energy accumulates deep within the Earth’s crust remains unclear and is one of the most fundamental open questions in seismological research. We investigate a quantitative link between earthquake radiated energy and the generalized Pourbaix electrochemical potential. This analysis forms the basis of a theoretical electrochemical framework for assessing whether electrical processes may contribute to earthquake nucleation. An intriguing similarity has been found between the released energy in an earthquake and Pourbaix potential in a redox reaction at an oxide-aqueous interface. A mathematical equivalence is established to strengthen this connection. This provides new insights into the possible electrochemical mechanism underlying seismic processes. Hydrated smectite, a clay mineral with a distinctive layered structure, is a dominant source of electrochemical potential generation in the Earth’s crust. Observations of significant smectite abundance in various deep drilling projects indirectly support this assertion. The layered arrangement of these hydrated clay minerals enables the formation of multiple electrochemical cells, leading to substantial accumulation of electrochemical potential. This observation indicates the presence of electrical potential in the earthquake preparation zone, which may offer a more comprehensive explanation for earthquake lights, negative anomalies in atmospheric electric fields, ionospheric perturbations, and other associated phenomena observed before or during an earthquake.

## Introduction

Earthquakes are among the most complex natural calamities, claiming human lives and causing extensive destruction to man-made structures. It is generally accepted that natural earthquakes occur due to the sudden slip or collision of tectonic plates within a fault zone deep in the Earth’s crust. The stored energy is released as seismic waves, producing ground motion and acceleration. With tremendous progress in modern science and instrumentation, seismological theory and instrumentation have become mature enough to provide quantitative source parameters rather than only magnitudes. A well-established moment magnitude ($M_w$) scale^1,2^ is used to quantify shallow and deep earthquakes based on radiated wave energy. This magnitude scale is derived from the concept of seismic moment ($M_0=\mu A D$), which is directly related to the measurable parameters in a seismic event, i.e., the rupture area (*A*) along the geological fault, the average displacement (*D*) or slip during the rupture, and the shear modulus ($\mu$) or elastic constant of the rock-forming material. The Richter–Gutenberg energy–moment magnitude relation^2^ for a seismic event is $\log E = 4.8 + 1.5M_w$, where *E* is the associated elastic energy in joules, that drives the earthquake from the hypocenter (focus), and $M_w$ is the moment magnitude of the earthquake. How does this tremendous amount of energy accumulate deep within the Earth’s crust? There are no clear clues to the origin of such energy accumulation deep within the Earth. Most seismic studies quantify parameters only after an actual seismic event occurs. The ultimate goal of earthquake research is to understand the nature of the seismic source and to enable the timely forecasting of impending earthquakes byidentifying suitable preseismic precursors.

Significant geochemical precursors, i.e., anomalous concentrations of dissolved ions^3^ and gases in groundwater, have been measured before intermediate and large earthquakes. Radon ($^{222}$Rn) anomalies in the environment (soil) and groundwater prior to earthquakes have been detected in many cases^4,5^. A wide range of pre-earthquake phenomena has been reported from both ground- and satellite-based observations. The atmosphere–ionosphere response a few days before the $M_w9$ Tohoku , Japan, earthquake revealed a rapid increase in outgoing long-wave radiation (OLR) in the infrared (10–13 $\upmu$m) and enhanced electron content (TEC) in the ionospheric region above the epicenter^6,7^. Thermal infrared (TIR) and ionospheric anomalies were detected in the Iran earthquake ($M_w5.8\sim 6.6$)^8^, the 2001 $M_w7.6$ Bhuj earthquake^9^, and the 2017 $M_w6.5$ Jiuzhaigou earthquake^10^. Preseismic anomalies in the telluric current (ATC) were observed on Kozu-shima Island, Japan, from 1997 to 2000^11^. Geoelectric potential changes are well documented in the literature as preseismic precursors^12–15^. Such preseismic anomalies are likely linked to the seismic electrical potential generated before or during earthquake rupture.

Recent studies have increasingly reported electrical, electromagnetic, and ionospheric anomalies preceding moderate to large earthquakes. Numerous case studies and statistical analyses have documented such signals in Japan, Mexico, Nepal, China, and other regions, indicating that electrical processes may be active during the earthquake preparation phase^16^. However, most reported anomalies are interpreted only as consequences of stress accumulation in fault zones, rather than as potential signatures of an independent energy source. This motivates examining whether the observed electrical signals are not merely stress-driven by-products but could indicate a stored energy source active during earthquake preparation.

While analyzing measurement data from an electrochemical sensor, a potential connection was identified between the Pourbaix electrochemical potential and the seismic electrical potential derived from earthquake-released energy. This observation suggests a strong resemblance between the estimated electrochemical potential in a solid-state electrode–aqueous system and the seismic electrical potential. It further motivated the investigation of the possible correlation between seismic electrical potential generated during earthquake events and the Pourbaix potential produced in an electrochemical reaction involving clay minerals and water. In this study a theoretical link between radiated seismic energy and the generalized Pourbaix electrochemical potential and evaluates whether this correspondence can arise from ion-exchange processes in hydrated clay minerals. We derive a mathematical equivalence, compare it with observed electrical signatures. The study proposes an electrochemical hypothesis that may help explain pre-seismic electrical anomalies and offers a framework for future validation.

## Methodology (theoretical framework)

### Derivation of equivalent electrical potential from released energy in an earthquake

Richter-Gutenberg energy magnitude relation accurately estimates seismic energy corresponding to the moment magnitude scale.

1 $$\begin{aligned} \begin{aligned} \log _{10} E&=4.8+1.5 M_w\\ E&=10^{4.8+1.5M_w} \text { (J)} \end{aligned} \end{aligned}$$

E is the released energy in joules and $M_w$ is the moment magnitude of an earthquake event. The magnitude $M_w = 4.0$ has the energy of $63.095 \times 10^{9}$ joules. The equivalent electrical potential was derived from the released energy using the following calculation. The electrical potential is calculated as follows.

2 $$\begin{aligned} \begin{array}{rcl} 63.095\times 10^9\,\textrm{J} & =& 0.01752\,\textrm{GWh} \\ & =& 0.01752\times 3600\times 10^{9}\,\mathrm {VA\cdot s} \\ & =& 0.01752\times 3600\times 10^{9}\,\mathrm {V\cdot C} \end{array} \end{aligned}$$

3 $$\begin{aligned} \begin{array}{rcl} 63.095\times 10^9\,\mathrm {J/C} & =& 0.01752\times 3600\times 10^{9}\,\textrm{V} \\ & =& 0.01752\times 3.6\times 10^{12} V\\ & =& \textrm{SEP}_{M_w=4.0} \times 3.6\times 10^{12}\,\textrm{V} \end{array} \end{aligned}$$

The Seismic Electrical Potential (SEP) increases exponentially with increasing magnitude . Other factors such as $3.6\times 10^{12}$ remain constant in the magnitude range $M_w (4.0\sim 5.9)$. Similarly, the equivalent electrical potential for other earthquake moment magnitudes is calculated using the same relation.

### Generalized Nernst-Pourbaix formulation and electrochemical potential at electrode-aqueous interfaces

The metal–oxide electrode responds to the aqueous pH buffer solution through a reversible electrochemical reaction. The redox electrochemical reaction occurring at the electrode –aqueous interface can be written as^17^,

4 $$\begin{aligned} M_{x}O_{y}+2yH^{+}+2ye^{-} = xM+yH_{2}O \end{aligned}$$

The electrode potential is calculated for the above reaction as follows,

5 $$\begin{aligned} E = E^{0} + \frac{0.0591}{2y} \log [a_{H^+}]^{2y} = E^{0} - 0.0591 \times pH \end{aligned}$$

where $E^0$ is the standard electrode potential and 0.0591 V is the Nernst slope at $25^{\circ }\textrm{C}$. A generalized Nernst equation was recently proposed^18^ based on Pourbaix’s pH –potential formulation, which was originally^19^ to describe corrosion equilibria; however, in this work we extend this thermodynamic framework to electrochemical sensors and to the electrode potentials generated at solid–aqueous interfaces.

We consider the general form^18,19^ of an electrode reaction as follows,

6 $$\begin{aligned} aA+mH^{+}+ne^{-} = bB+cH_{2}O \end{aligned}$$

in which A represents a simple metallic ion or metallic oxide, and B is the corresponding metal. The electrode potential is calculated for the above reaction as follows,

7 $$\begin{aligned} \begin{aligned} E&= E^0 - \frac{0.0591}{n} \log \frac{(a_B)^b (a_{H_2O})^c}{(a_A)^a (a_{H^+})^{m}} \\&= E^0 + 0.0591 \left( \frac{m}{n} \right) \log (a_{H^+}) - \frac{0.0591}{n} \log \frac{(a_B)^b (a_{H_2O})^c}{(a_A)^a} \end{aligned} \end{aligned}$$

Assuming the activity of A, B, and $H_2O$ unity, the equation is expressed as follows:

8 $$\begin{aligned} E = E^0 + 0.0591 \left( \frac{m}{n} \right) \log (a_{H^+}) = E^0 - 0.0591 \times x \times \text {pH} \end{aligned}$$

The Nernst potential of 0.0591 V $pH^{-1}$ can be assigned as NP, which is a special case of Equation (9) owing to the symmetric ion-exchange factor $(x=1)$. The electrode potential arising from asymmetric ion-exchange ($x\ne 1$) can be assigned as the Pourbaix Potential, or PP, as the idea of asymmetric ion exchange was originally proposed by M. Pourbaix through the pH–potential formulation^19^. A similar non-Nernstian higher-order ion-exchange formulation in ionophore-based ion-sensitive electrodes was reported by Shigeru et al. and Bakker et al.^20,21^. An equation linking the PP and NP is given as follows:

9 $$\begin{aligned} PP_x=NP\times x \end{aligned}$$

The Pourbaix Potential (PP) expressed in Eq. (9) is quantitatively compared with the radiated seismic electrical potential in the result section “Extrapolated Pourbaix potential and quantitative comparison with seismic electrical potential (SEP)”. This comparison forms the basis of a quantitative bridge between the Seismic Electrical Potential (SEP) and the Pourbaix Potential (PP).

### Mathematical derivation of the equivalence between the Nernst–Pourbaix potential and radiated seismic energy

To establish a connection between earthquake energy and the generalized Nernst equation (Pourbaix Equation), the terms of both equations are rearranged as shown below^1,2^.

10 $$\begin{aligned} \begin{aligned} log E_{EQ}&=4.8+1.5M_w\\ \Rightarrow M_w&=\frac{2}{3}logM_0-10.7\\ \Rightarrow log E_{EQ}&=-11.25+logM_0 \end{aligned} \end{aligned}$$

The relation between moment magnitude ($M_w$) and seismic moment ($M_0$) is introduced here. $E_{EQ}$ denotes the energy released during an earthquake. The suffixes (EQ – earthquake and ECR – electrochemical reaction) are used in the equations for clarity regarding the source of energy. Now we consider the generalized Nernst-Pourbaix equation (Eq. 8)^18^,

11 $$\begin{aligned} E_{ECR}-E_{0} = -k\times x \end{aligned}$$

where $E_{ECR}$ is the electrode potential, $E_0$ is the standard (reference) electrode potential and $k = 0.059 \times pH$. If the reference electrode potential $E_0$ is set to 0, it becomes

12 $$\begin{aligned} E_{ECR} =-k \times x \end{aligned}$$

Taking the modulus of both sides, the equation becomes

13 $$\begin{aligned} \mid E_{ECR} \mid =\mid -k\times x\mid \end{aligned}$$

Taking the logarithm of both sides, the equation becomes

14 $$\begin{aligned} log \mid E_{ECR} \mid =log(k)+log(x) \end{aligned}$$

For $pH = 1$, the above equation becomes

15 $$\begin{aligned} log E_{ECR} =log(0.059)+log(x) \end{aligned}$$

Putting two equations (Eqs. 10 and 15) together for comparison,

16 $$\begin{aligned} log E_{EQ}=-11.25+logM_0 \end{aligned}$$

17 $$\begin{aligned} log E_{ECR}= -1.229+log(x) \end{aligned}$$

Equations (16) and (17) are mathematically identical. This mathematical equivalence enables a direct quantitative comparison between *PP*–*x* and *SEP*–$M_{0}$ which will be discussed in result section “Scaling relationship linking seismic electrical potential and Pourbaix potential”.

### Nature of data and study scope

This study does not utilize earthquake catalog data, field measurements, or observations from a specific geographic region. All quantities analyzed in this work–including radiated seismic energy, moment magnitude, and the electrochemical Pourbaix potential–are derived from well-established theoretical relations reported in the literature. The energy–magnitude relation and the Pourbaix electrochemical formulation are treated analytically to examine their mathematical equivalence. Because the objective of this work is to develop a theoretical link between radiated seismic energy and electrochemical potential, no study-area–specific datasets are employed. The findings are hypothesis-driven theoretical framework rather than an analysis of region-specific earthquake records.

## Results

### Computed seismic electrical potential (SEP) for different moment magnitudes

Table 1 shows the details of earthquake magnitude $M_w$ ($4.0\sim 5.9$) and corresponding seismic electrical potentials. The magnitude range $M_w$ ($4.0\sim 5.9$) is used as an arbitrary set for calculation purposes. Other sets –$M_w$ ($0.0\sim 1.9$), $M_w$ ($2.0\sim 3.9$), $M_w$ ($6.0\sim 7.9$), and $M_w$ ($8.0\sim 9.9$) [Table S1–S4]– follow the same trend as $M_w$ ($4.0\sim 5.9$). The electrical potential linked to earthquakes and the potential produced by spontaneous electrochemical reactions will be explored in the next section.

**Table 1:** Details of Earthquake energy in joules, watt-hours, and the corresponding seismic electrical potential (SEP) (joule per coulomb).

$M_w$	$\text {Energy GJ}(\times 10^9\text {J})$	$\text {GWh}(\times 10^9\text {Wh})$	$\text {SEP}\times 3.6\times 10^{12} \text {V}$
4.0	63.095	0.0175	0.0175
4.1	89.125	0.024	0.024
4.2	125.892	0.035	0.035
4.3	177.827	0.049	0.049
4.4	251.188	0.069	0.069
4.5	354.813	0.098	0.098
4.6	501.187	0.139	0.139
4.7	707.945	0.196	0.196
4.8	1000	0.277	0.277
4.9	1412.537	0.392	0.392
5.0	1995.262	0.554	0.554
5.1	2818.382	0.783	0.783
5.2	3981.071	1.106	1.106
5.3	5623.413	1.562	1.562
5.4	7943.282	2.206	2.206
5.5	11220.184	3.116	3.116
5.6	15848.931	4.402	4.402
5.7	22387.211	6.218	6.218
5.8	31622.776	8.784	8.784
5.9	44668.359	12.408	12.408

### Extrapolated Pourbaix potential and quantitative comparison with seismic electrical potential (SEP)

The abundance of phyllosilicates, which consist predominantly of $SiO_2$ and $Al_2O_3$ in the Earth’s crust, directs attention toward materials containing trivalent and tetravalent elements and their associated electrochemical potentials in aqueous solutions involving different ion-exchange factors.Various experimental electrochemical potentials for $Al_2O_3$ and $SiO_2$ have been reported in the literature. The minimum value for $Al_2O_3$ was 17 mV^22^ for low-temperature grown oxide, whereas the ideal value is expected to be 40–55 mV^23^. Reddy et al.^24^ reported 30 mV for their porous silicon (P-Si) structure. Naif et al.^25^ reported 66 mV in a similar P-Si sensing electrode. Larry et al.^26^ reported 108 mV for a porous silicon quantum dot probe. Based on the Pourbaix formulation, the ideal $Si\text {--}SiO_2$ system could exhibit electrode potentials of 59.16 mV, 73.9 mV, and 88.6 mV depending on the ion-exchange factors $x = 4/4$, $x = 5/4$, and $x = 6/4$, respectively. A very high electrode potential of 300 mV was achieved in a 3D nanoporous P-Si structure^27^, which could be attributed to an ion-exchange factor of $x = 21/4$ $(PP_{21/4} = 310\ \textrm{mV})$. Table 2 shows the reported experimental electrochemical potentials of the metal-oxide–aqueous interface and the corresponding higher-order ion-exchange factors. The trend of increasing electrode potential from 30 mV to 300 mV for a unit electrochemical cell in the $Si\text {--}SiO_2$ system indicates that the potential may be considerably higher when higher-order ion exchange occurs at the interfaces.

**Table 2:** Details of experimental electrochemical potential obtained from $SiO_2(\Delta Z=+4)$, $Al_2O_3(\Delta Z=+3)$ and other trivalent-tetravalent element systems.

Material	$PP_x$[Expt.](V)	$\Delta Z$	*x*	$PP_x$[Th.](V)	References
$SiO_2$	0.030	+ 4	$\frac{3}{4}$	0.044	^24^
$SiO_2$	0.066	+ 4	$\frac{5}{4}$	0.073	^25^
$SiO_2$	.......	+ 4	$\frac{6}{4}$	0.088	^19^
$SiO_2$	0.108	+ 4	$\frac{8}{4}$	0.118	^26^
$SiO_2$	0.300	+ 4	$\frac{21}{4}$	0.310	^27^
$Al_2O_3$	0.017	+ 3	$\frac{1}{3}$	0.019	^22^
$Al_2O_3$	0.055	+ 3	$\frac{3}{3}$	0.059	^23^
$Al_2O_3$	.......	+ 3	$\frac{4}{3}$	0.078	^19^
$Ga_2O_3$	0.079	+ 3	$\frac{5}{3}$	0.098	^28^
$Ga_2O_3$	0.092	+ 3	$\frac{5}{3}$	0.098	^18^
$Ga_2O_3$	0.110	+ 3	$\frac{6}{3}$	0.118	^18^
$Ir_2O_3$	0.069	+ 3	$\frac{4}{3}$	0.078	^29^
$Ir_2O_3$	0.115	+ 2	$\frac{4}{2}$	0.118	^30^

**Table 3:** Details of earthquake electrical potential (joule per coulomb), electrochemical potential at $25^\circ \!C$ from redox reaction of trivalent and tetravalent elemental oxide with different ion-exchange factors.

$M_w$	SEP	x($\Delta Z=+3$)	$PP_x$(V)	x($\Delta Z=+4$)	$PP_x$(V)
4.0	0.0175	$\frac{1}{3}$	0.0197	$\frac{1}{4}$	0.0147
4.1	0.024	$\frac{1}{3}$	0.0197	$\frac{2}{4}$	0.0295
4.2	0.035	$\frac{2}{3}$	0.0394	$\frac{3}{4}$	0.0443
4.3	0.049	$\frac{3}{3}$	0.0591	$\frac{4}{4}$	0.0591
4.4	0.069	$\frac{4}{3}$	0.0788	$\frac{5}{4}$	0.0739
4.5	0.098	$\frac{5}{3}$	0.0986	$\frac{7}{4}$	0.1035
4.6	0.139	$\frac{7}{3}$	0.1380	$\frac{9}{4}$	0.133
4.7	0.196	$\frac{10}{3}$	0.1972	$\frac{13}{4}$	0.1922
4.8	0.277	$\frac{14}{3}$	0.2760	$\frac{19}{4}$	0.2810
4.9	0.392	$\frac{20}{3}$	0.3944	$\frac{27}{4}$	0.3993
5.0	0.554	$\frac{28}{3}$	0.5521	$\frac{38}{4}$	0.5620
5.1	0.783	$\frac{40}{3}$	0.7888	$\frac{53}{4}$	0.7838
5.2	1.106	$\frac{56}{3}$	1.1043	$\frac{75}{4}$	1.1092
5.3	1.562	$\frac{79}{3}$	1.5578	$\frac{106}{4}$	1.5677
5.4	2.206	$\frac{112}{3}$	2.2086	$\frac{149}{4}$	2.2037
5.5	3.116	$\frac{158}{3}$	3.1157	$\frac{211}{4}$	3.1206
5.6	4.402	$\frac{224}{3}$	4.4172	$\frac{298}{4}$	4.4074
5.7	6.218	$\frac{315}{3}$	6.2118	$\frac{420}{4}$	6.2118
5.8	8.784	$\frac{445}{3}$	8.7754	$\frac{594}{4}$	8.7852
5.9	12.408	$\frac{629}{3}$	12.4038	$\frac{839}{4}$	12.4088

**Fig. 1:**
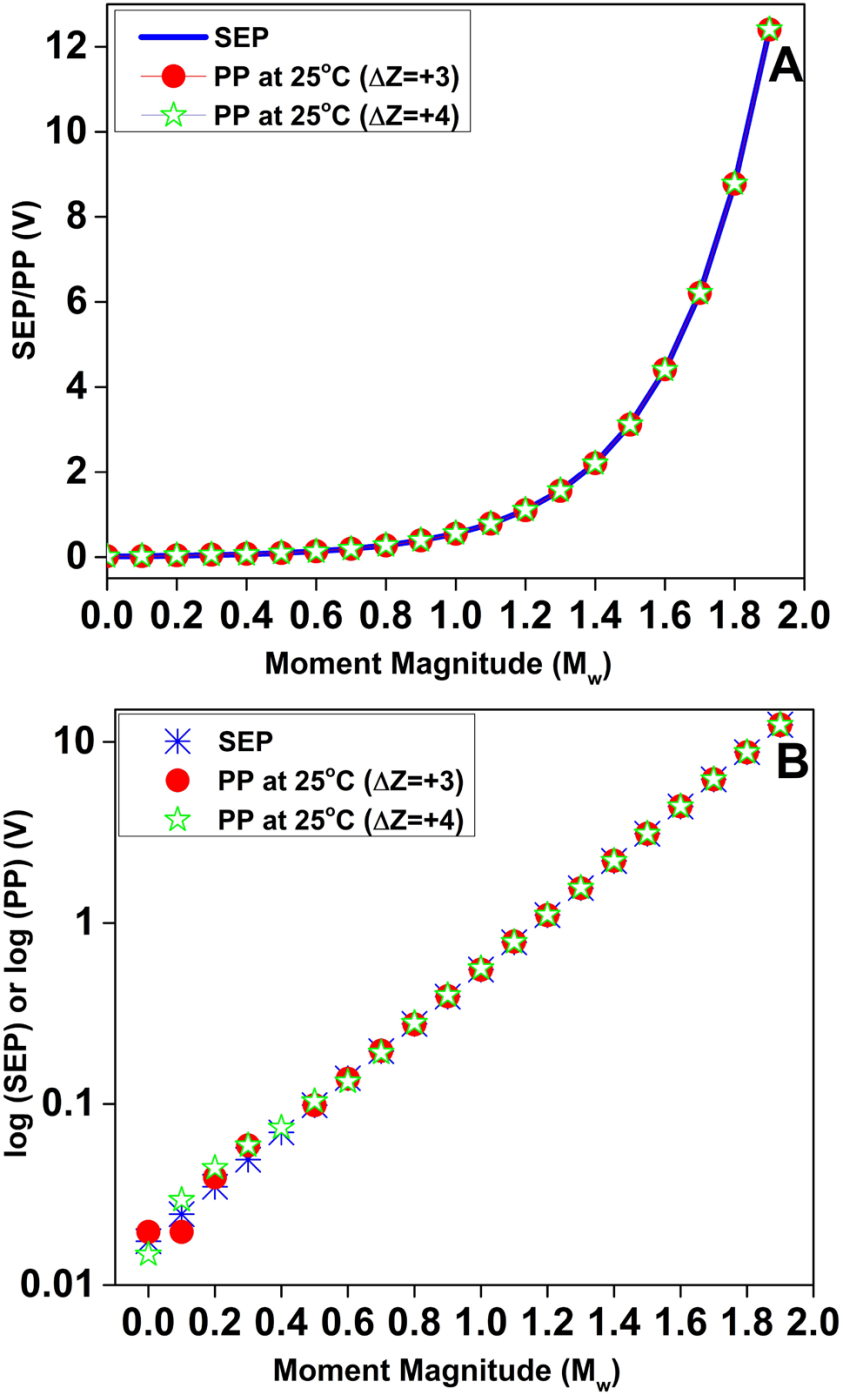
Correlation between Seismic Electrical Potential (SEP) and Pourbaix Potential (PP). (**A**) Seismic Electrical Potential (SEP) and the corresponding Pourbaix Potential (PP), evaluated at $25^{\circ }$C, plotted as functions of earthquake moment magnitude $M_w$ in the range $4.0 \le M_w \le 5.9$. SEP is derived directly from the radiated seismic energy scaling with $M_w$, whereas PP is obtained from the generalized Nernst–Pourbaix formulation through the ion-exchange factor *x*. The close quantitative correspondence between SEP and PP across this magnitude range indicates that variations in seismic energy with $M_w$ can be consistently mapped onto variations in electrochemical potential governed by *x*. (**B**) Semi-logarithmic plot of SEP/PP versus earthquake moment magnitude $M_w (4.0\sim 5.9)$ in which the linear trend follows $\log (\textrm{SEP}) = A + B\,M_w$, consistent with the Gutenberg–Richter energy–magnitude relation $\log E = 4.8 + 1.5 M_w$ (Eq.1).

Based on the experimental values of electrode potential in $Si-SiO_2$ and $Al-Al_2O_3$ systems and Pourbaix’s formulation of higher-order ion-exchange(i.e., m/n ratio) in several materials systems ($Ga-Ga_2O_3$, $Ir-Ir_2O_3$, $V-V_2O_3$, $Mn-Mn_3O_4$, $Ni-Ni_3O_4$, $Cr-Cr_2O_3$, $Cr-Cr_3O_4$ )^19^, It is reasonable to generalize the Pourbaix Potential (PP) for any material system. The values can be extrapolated from the trend of higher-order ion exchange by considering the chemisorption of increasing amounts of hydrogen ion ($H^+$) at the electrode–aqueous interface. Table 3 shows the Pourbaix potentials at different ion-exchange factors that closely match the corresponding SEP values for $M_w = 4.0\sim 5.9$. To understand the correlation between SEP and PP, both SEP (V) vs. $M_w (4.0\sim 5.9)$ and $\log (\textrm{SEP})$ vs. $M_w (4.0\sim 5.9)$ curves (Fig. 1A and B) are plotted. The theoretical temperature dependence of the electrode potential is 0.02 mV per kelvin^31^. Considering a temperature of $200^{\circ }C$ at 75 km depth^32^ in the Earth’s crust, the change in electrode potential is $\Delta T\times 0.02\ \textrm{mV} = 175\times 0.02\ \textrm{mV} = 3.5\ \textrm{mV}$, which is very small and does not significantly alter the original Pourbaix potential calculated at $298.15\ \textrm{K}\ (25^{\circ }C)$. The change in PP from $PP_{1/4} = 0.01725\ \textrm{V}$ $(PP_{1/3} = 0.01972\ \textrm{V})$ to $PP_{839/4} = 12.408\ \textrm{V}$ $(PP_{629/3} = 12.403\ \textrm{V})$

corresponds to increasing ion-exchange factors. Although PP increases approximately linearly with the ion-exchange factor *x*, its comparison with seismic electrical potential (SEP) appears exponential because moment magnitude ($M_w$) is defined on a logarithmic energy scale. The exponential adsorption kinetics is a well-established phenomenon in solid–liquid^33–36^ and solid–gas^37–39^ interfaces, which governs the chemisorption process involving multilayer adsorption sites. The apparent exponential similarity between SEP and PP arises not from the intrinsic chemistry of PP, but from the logarithmic definition of earthquake magnitude. The quantitative similarity between SEP and Pourbaix potential (PP) is intriguing, and identifying a physical mechanism that may inherently link these two parameters requires further investigation.

### Scaling relationship linking seismic electrical potential and Pourbaix potential

A quantitative equivalence between SEP and PP is discussed through detailed analysis in previous sections. An underlying physical principle may connect the two equations, which requires additional analysis. The two energies $(E_{EQ}, E_{ECR})$ are linked with a constant factor. To allow direct comparison with the seismic relation, we denote the electrochemical energy term $E_{ECR}$ as the Seismic Electrical Potential (SEP), representing the energy-per-unit-charge associated with redox-driven charge buildup in the fault zone. In the case of $M_w (4.0\sim 5.9)$, the constant scaling factor is $3.6\times 10^{12}$. The following equation shows the relation where $E_{EQ}$ is the equivalent electrical potential released in the $M_w (4.0\sim 5.9)$ range, and $E_{ECR}$ is the electrical potential generated in an electrochemical reaction. Moment magnitude $M_w (4.0\sim 5.9)$ and corresponding $E_{ECR}$ (SEP) are presented in Table 1. They are linked with the following relation,

18 $$\begin{aligned} E_{EQ}=3.6\times 10^{12}E_{ECR} \end{aligned}$$

Taking the logarithm of both sides and substituting the terms from Eqs. (17) and (18),

19 $$\begin{aligned} \begin{aligned} log (E_{EQ})=log(3.6\times 10^{12})+log(E_{ECR})\\ \Rightarrow -11.25+log M_o=0.556+12-1.229+log(x)\\ \Rightarrow log M_o=log (x)+22.577\\ \Rightarrow log(\frac{M_0}{x})=22.577\\ \Rightarrow \frac{ M_0}{x}=10^{22.57} \end{aligned} \end{aligned}$$

**Fig. 2:**
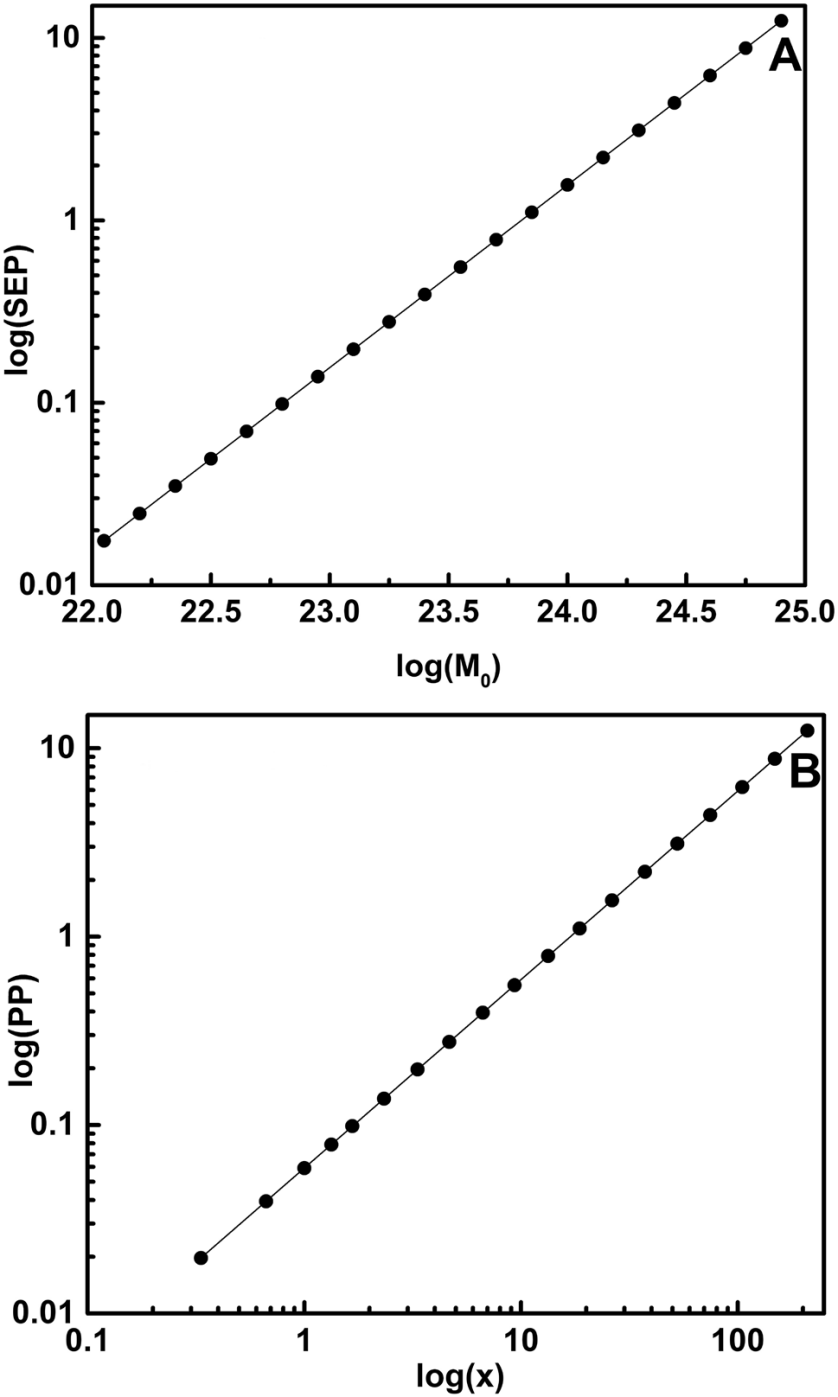
Correlation between Seismic Electrical Potential (SEP) and Pourbaix Potential (PP). (**A**) Log–log dependence of the Seismic Electrical Potential (SEP) on earthquake seismic moment $M_0$ $(4.0 \le M_w \le 5.9)$, following the relation $\log (\textrm{SEP}) = -23.811 + \log (M_0)$. (**B**) Log–log dependence of the Pourbaix Potential (PP) on the ion–exchange factor *x*, described by $\log (\textrm{PP}) = -1.228 + \log (x)$. The linear trends in both cases demonstrate a direct mathematical correspondence between seismic energy scaling and electrochemical potential scaling.

The $M_0$ and x are linked through a scaling factor of $10^{22.57}$. This suggests that both equations describe the same underlying physical phenomenon: earthquake nucleation driven by energy accumulation, where ion exchange plays a crucial role in electrochemical energy generation. Based on the data points from Table 3, the log (SEP) vs. log ($M_0$) and log (PP) vs. log (x) are plotted as shown in Fig. 2. Both characteristics are linear. A linear fit function results in the following equations,

20 $$\begin{aligned} log(SEP)=-23.811+log(M_0) \end{aligned}$$

21 $$\begin{aligned} log(PP)= -1.228+log(x) \end{aligned}$$

As SEP and PP are quantitatively equal, the relation between $M_0$ and *x* is as follows:

22 $$\begin{aligned} \frac{ M_0}{x}=10^{22.58} \end{aligned}$$

The scaling factor calculated analytically matches the value derived from the graph, validating the consistency between the theoretical and graphical approaches. Although $10^{22.5}$ characterizes the $M_{w}\,4.0$–5.9 range, the same proportionality holds for other magnitude intervals, with the exponent shifting by approximately ±3 (e.g., $\sim 10^{25.5}$ for $M_{w}\,6.0$–7.9, $\sim 10^{28.5}$ for $M_{w}\,8.0$–9.9, and $\sim 10^{19.5}$ for $M_{w}\,2.0$–3.9), indicating scale-dependent correspondence between seismic energy and electrochemical potential.

## Discussion

### Observed electrical signatures associated with earthquakes

It is generally accepted that an earthquake is a mechanical phenomenon. However, abundant evidence suggests the presence of electrical signatures^16,40–56^ associated with earthquakes. Variations in the atmospheric electric field preceding earthquakes have been reported in many studies^13,57^. Lithosphere–atmosphere–ionosphere coupling (LAIC) is a well-known framework for understanding the perturbation in the ionosphere ionospheric perturbations. Variations in ionospheric total electron content (TEC) have been observed before many earthquakes ($M_w>5.0$) through analyses of global positioning satellite data. The appearance of geoelectric voltage in seismically active regions is believed to be one of the possible sources of such ionospheric disturbances^58^. Pulinets et al.^59^ proposed that large-scale, high-intensity vertical electric fields appearing in a seismically active region a few days before a mainshock can penetrate the ionosphere and generate ionospheric anomalies. TEC anomalyies are also observed above large thunderstorms, whereas no detectable localized TEC variation is observed during a thunderstorm-quiet night^60^.

Earthquake lights (EQLs) are atmospheric optical phenomena observed before, during, and sometimes after earthquakes^61,62^. The enhancement of electrical fields in the Earth’s crust and lightning-like discharges have been for EQLs^63^. Some studies have even suggested that electrical discharges may trigger earthquakes^64–66^. Only electrical discharges and their associated shock waves can propagate over large distances at comparable speeds to seismic waves, reinforcing the possibility of rapid underground electrical phenomena. Taken together, electromagnetic, atmospheric, and ionospheric observations indicate that measurable electric fields often accompany the earthquake preparation phase. These signals are not readily explained solely by mechanical failure models and may reflect charge generation and transport within the crust. From an electrochemical perspective, such electrical activity can arise from asymmetric ion exchange and charge accumulation at hydrated clay mineral interfaces within clay-rich fault zones.

This mechanism offers a potential physical interpretation connecting subsurface electrochemical processes to geoelectric signals, atmospheric anomalies, and ionospheric perturbations detected before large earthquakes. In several articles, phenomena associated with earthquakes are described as “underground thunderstorms” or “underground lightning,”^67^ suggesting the occurrence of sudden, large-scale electrical discharges beneath the Earth’s surface. From this electrochemical perspective, this analogy can be grounded in real physical processes: the accumulation and separation of electrochemical charges at clay mineral interfaces, particularly within clay-rich and fluid-bearing fault zones, can generate significant electric fields over time. All these electrical signatures associated with seismic activity provide clear hints into the complex processes of electrical charge accumulations in earthquake preparation zones.

### Comparison with electrokinetic models

Electrokinetic models^68,69^ attribute preseismic electrical signals to streaming potentials produced by pressure-driven fluid flow in permeable rocks. Electrokinetic models explain electrical anomalies in high-permeability faults, their applicability is limited in clay-rich gouges, where permeabilities^70^ can fall to $10^{-18} \sim 10^{-21}$ m$^{2}$, making sustained fluid flow unlikely. In these low-permeability settings, our hypothesis provides a complementary mechanism: electrochemical charge accumulation arising from asymmetric ion exchange within hydrated clay layers. Because this reaction-based process does not require fluid transport and it may help explain electrical anomalies observed before earthquakes in clay-dominated faults. Thus, electrokinetic and electrochemical pathways may operate under different geological conditions, offering a broader interpretation of earthquake-related electrical anomalies.

### Electrochemical potential generation in clay minerals: a proposed mechanism

The extrapolated electrochemical potential (Pourbaix potential) obtained from the redox reactions of $Al_2O_3$ ($\Delta Z=+3$) and $SiO_2$ ($\Delta Z=+4$) with aqueous pH buffer at different ion-exchange factors closely matches the seismic electrical potential (SEP) (Table 3). Naturally, it is important to focus on the sources of $SiO_2$ and $Al_2O_3$ in the Earth’s crust and within earthquake fault zones. In nature, during the weathering process, rocks react with water and form clay minerals. Clay minerals are phyllosilicates, consisting of stacked two-dimensional (2D) sheets of hydrated aluminosilicates $(SiO_2, Al_2O_3, H_2O)$ found in geological deposits, terrestrial weathering environments, and marine sediments^71^. Water is present in varying amounts as part of the mineral structure. Based on the stacking of silicon tetrahedra (T) and alumina octahedra (O) sheets within the crystal layers, clay minerals are categorized into three groups: 1T:1O, 2T:1O, and 2T:2O types. Clay minerals have a wide range (60-120 meq / 100 g) of cation-exchange capacities^72^ due to the overall charge imbalance in the crystal lattice.

**Fig. 3:**
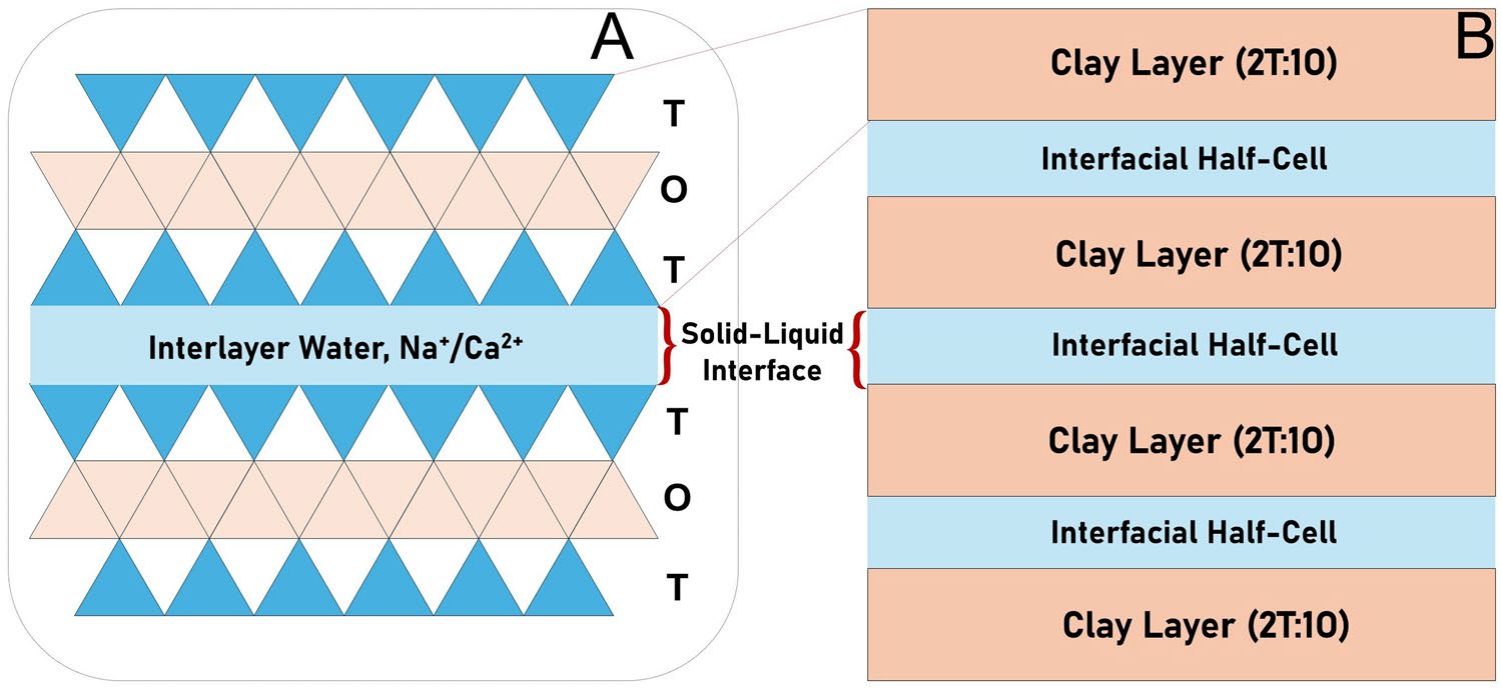
Schematic representation of (**A**) a typical 2T:1O smectite clay layer structure showing the interlayer water and exchangeable cations sharing two solid–liquid interfaces. (**B**) Formation of interfacial half-cells at each interface in layered smectite clay leading to charge accumulation.

The fascinating clay mineral structure, with attractive and large surface areas^73^, allows interfacial reactions in aqueous environments. In general, it is estimated that a cubic centimeter (1 cm$^{3}$) of clay has a reactive surface of around 2800 m$^{2}$, equivalent to the area of a football field. For an analogy, a centimeter-thick pad of paper includes about 100 sheets, whereas a centimeter-thick layer of clay minerals includes about 100,000 sheets. Such a enormous number of clay sheets gives a sense of the number of electrochemical cells^74^ required to produce a enormous electrical potential equivalent to earthquake energy. Figure 3a shows a typical smectite clay layer structure and the existence of interlayer water and exchangeable cations. Two solid-liquid interfaces are available in each unit cell for electrochemical reactions. Figure 3b shows the possibility of forming interfacial half-cells in each interlayer of a stacked clay. Smectite, vermiculite, and illite are ideal candidates for generating such significant electrochemical potentials owing to the large cation exchange capacity in the interlayer spacing and outstanding adsorption properties. Dissociative chemisorption of water^75^ on silica surface and formation of hydronium ion could explain the increasing number of hydrogen ion adsorption on the clay surface.

In an notable connection, smectite clay is predominantly found in various scientific deep drilling projects^76–81^. The Taiwan Chelungpu Fault Drilling Project (TCDP) revealed a spike in smectite^76^, decreases in illite, and the disappearance of chlorite and kaolinite in the primary slip zone (PSZ). Integrated Ocean Drilling Program Expedition 343 (Japan Trench Fast Drilling Project, JFAST) was carried out one year after the 2011 Tohoku-Oki earthquake ($M_w 9.0$). The mineralogical analyses revealed that the shallow portion of the megathrust is enriched in smectite (60–80 wt.%) compared with the surrounding sediments^80^. Scientific drilling of the San Andreas Fault also revealed high concentrations of the clay mineral smectite^81^.The inherent weakness and low -friction nature of clay minerals in fault zones presents a profound paradox: How can such mechanically weak materials accumulate and sustain the elastic strain energy required to trigger large, destructive earthquakes? This apparent contradiction challenges conventional fault mechanics. Recent investigations^82–86^, including studies on the seismic potential of weak, near-surface faults at plate -tectonic slip rates, and laboratory and temperature-based analyses of the Tohoku-Oki megathrust fault, have provided valuable insights into the ability of weak faults to host large ruptures under low shear-stress conditions. However, despite these advances, a complete understanding of the mechanisms enabling energy accumulation and sudden release in clay -rich fault zones remains elusive. These observations indicate that mechanical processes alone may be insufficient, motivating further exploration of additional mechanisms–such as electrochemical energy buildup within clay-water interfaces–that may contribute to earthquake nucleation.

### Feasibility of electrochemical energy storage in Clay-Rich fault zones

The feasibility of generating seismic-scale energy through electrochemical processes arises naturally when the clay-rich fault zone is viewed as an enormous distributed charge–potential system. At the interface, the generalized Nernst–Pourbaix relation sets the voltage per unit charge (PP), which takes the form $E_{\textrm{ECR}} = PP_x \times \textrm{pH}$ when higher-order ion exchange and realistic pore-fluid pH values (7–10) are considered, representing the energy available per coulomb in each nanoscale clay–water electrochemical cell. The total electrochemical energy stored in the fault follows the fundamental relation $E = QV$, where the cumulative charge $Q$ is supplied by the vast cation-exchange reservoir of smectite^72^. A single cubic centimeter of smectite contains thousands of square meters of reactive surface area^73^, and its typical cation-exchange capacity of 60–120 meq/100 g corresponds to approximately $10^{19}$–$10^{21}$ ion-exchange sites. Crucially, the large number of ion-exchange sites represents fixed structural charge rather than freely mobile charge. Despite this enormous site density, only a small fraction can be converted into usable free charge through electrochemical charge-separation processes. When aggregated over large fault–zone volumes, clay-rich material containing vast numbers of interlayer domains can act collectively as a network of parallel nano-batteries. The resulting charge reservoir becomes sufficiently large that, when multiplied by volt-scale values of $V_{\textrm{ECR}} = PP_x \times \textrm{pH}$ (typically 1–100 V), even modest fault-zone volumes can yield total energies $E = QV$ on the order of $10^{10}$–$10^{13}$ J, comparable to the radiated seismic energy of $M_w$ 4–6 earthquakes. In this framework, PP does not supply the total earthquake energy; rather, PP defines the energy-per-unit-charge, while the massive clay-hosted charge reservoir provides the multiplicative factor required to reach $10^{10}$–$10^{13}$ J. The quantitative equivalence between SEP and PP identified in this study supports the physical plausibility of a mechanism in which electrochemical charge separation accumulates energy over long timescales, and mechanical rupture emerges as a secondary release pathway once electrical instability overwhelms a weakened clay-rich fault zone.

### Limitations

The present study establishes a quantitative similarity between radiated seismic energy and the generalized Pourbaix electrochemical potential using normalized energy expressions. This normalization requires assigning $Q = 1$ C in the relation $E = QV$, which defines an energy-per-unit-charge scale without prescribing the actual charge stored in a fault zone. While this approach is dimensionally consistent, the scaling constant ($3.6\times 10^{12}$ in the Gutenberg–Richter relation) represents only the radiated fraction of total earthquake energy and does not capture other dissipative pathways.

Likewise, the ion-exchange factor $x=m/n$ expresses redox energetics at a solid–liquid interface but does not explicitly model kinetic constraints, spatial heterogeneity, or multi-stage charge buildup in natural clay minerals. Thus, the present results outline a plausible mechanism that can be strengthened through targeted laboratory measurements and integrated geophysical monitoring. Further laboratory experiments and combined geophysical observations are needed to test whether this electrochemical process actually contributes to earthquake-related electrical signals.

In addition, the derived correspondence between seismic moment and ion-exchange factor, expressed through the relation $M_{0}/x = 10^{22.5}$ for the $M_{w}=4.0$–5.9 interval, reflects a magnitude-dependent scaling. This proportionality shifts by approximately three orders of magnitude across adjacent magnitude ranges (e.g., $M_{w}=2$–3.9 or $M_{w}=6$–7.9), indicating that the $M_{0}$–*x* relationship is a mathematical normalization rather than a direct physical mapping.

## Conclusion

A quantitative link between radiated seismic energy and electrochemical potential is established in this study through a mathematical equivalence between the earthquake energy relation and the generalized Nernst–Pourbaix formulation. This equivalence demonstrates that electrochemical energy generated by asymmetric ion exchange at hydrated clay–water interfaces can scale consistently with earthquake radiated energy. The result identifies electrochemical charge separation as a viable and previously unquantified energy reservoir within fault zones. These findings suggest that energy accumulation in earthquakes need not arise exclusively from mechanical strain alone. Instead, electrochemical processes operating within clay-rich fault cores may provide the primary energy buildup, with mechanical rupture representing a subsequent mode of energy release. In this view, elastic failure, frictional slip, and seismic wave propagation emerge as secondary manifestations of an underlying electrical instability. The proposed electrochemical hypothesis offers a unified physical framework capable of explaining both earthquake energy scaling and the diverse electrical, electromagnetic, atmospheric, and ionospheric anomalies reported prior to major seismic events. By identifying a common electrochemical origin rooted in fault-zone mineralogy and interfacial ion exchange, this work points toward a coherent mechanism linking subsurface energy accumulation and observable pre-seismic electrical signatures. While further experimental and geophysical validation is required, the framework presented here establishes a theoretical foundation for reinterpreting earthquakes as fundamentally electrochemical in origin. This perspective has the potential to reshape the understanding of earthquake nucleation and to guide future efforts aimed at detecting and quantifying electrical precursors to seismic failure.

## Supplementary Information

Supplementary Information.

## Data Availability

All data are reported in the paper or supplementary materials.
