# Supplementary material for: An electrochemical hypothesis of earthquakes exploring a theoretical link between radiated seismic energy and Pourbaix potential
Source: Sci Rep. 2026 Feb 17;16:8701. doi: 10.1038/s41598-026-40629-w (PMC12979594; doi:10.1038/s41598-026-40629-w)
Supplement: Supplementary file 1 — Supplementary Information. [file 41598_2026_40629_MOESM1_ESM.pdf]

An Electrochemical Hypothesis of Earthquakes Exploring a Theoretical Link Between Radiated Seismic Energy and Pourbaix Potential Supplementary Material

Atanu Das[1,*] and Sankar Prasad Bag[2]

[1]Manipal Institute of Technology, Manipal Academy of Higher Education, Manipal-576104, India
[2]School of Electronics and Communication Engineering, REVA University, Bangalore-560064, India
[*]Corresponding author: atanu.das@manipal.edu

ABSTRACT

This supplementary document provides detailed derivations and tabulated calculations used to obtain the seismic electrical potential (SEP) from earthquake radiated energy across different magnitude ranges.

Results

1 Derivation of Equivalent Electrical Potential from Released Energy in an Earthquake

Richter–Gutenberg energy magnitude relation accurately estimates seismic energy corresponding to the moment magnitude scale.

$$\begin{aligned} \log_{10} E &= 4.8 + 1.5M_w \\ E &= 10^{4.8+1.5M_w}\,\mathrm{J} \end{aligned} \tag{1}$$

E is the released energy in joules and M_w is the moment magnitude of an earthquake event. The magnitude $M_w = 4.0$ has the energy 63.095×10^9 J.

The equivalent electrical potential is obtained as

$$\begin{aligned} 63.095 \times 10^9\,\mathrm{J} &= 0.01752\,\mathrm{GWh} \\ &= 0.01752 \times 3600 \times 10^9\,\mathrm{V\,C} \end{aligned} \tag{2}$$

$$\begin{aligned} 63.095 \times 10^9\,\mathrm{J/C} &= 0.01752 \times 3.6 \times 10^{12}\,\mathrm{V} \\ &= \mathrm{SEP}_{M_w=4.0} \times 3.6 \times 10^{12}\,\mathrm{V} \end{aligned} \tag{3}$$

The Seismic Electrical Potential is denoted as SEP, which only increases exponentially with the increasing magnitude scale. Other factors, i.e., 3.6×10^{12} will remain constant in the magnitude range $M_w(4.0 \sim 5.9)$. Similarly, the equivalent electrical potential for other earthquake moment magnitudes can be calculated. Table 1. shows details of earthquake magnitude M_w $(4.0 \sim 5.9)$ and corresponding seismic electrical potentials. The magnitude scale M_w $(4.0 \sim 5.9)$ is considered an arbitrary set for calculation. Other sets[Table S1-S4] like M_w $(0.0 \sim 1.9)$, M_w $(2.0 \sim 3.9)$, M_w $(6.0 \sim 7.9)$, and M_w $(8.0 \sim 9.9)$ will follow the same trend as M_w $(4.0 \sim 5.9)$.

Table 1. Details of Earthquake energy in joule, watt-hour, and equivalent seismic electrical potential (SEP) (joule/coulomb)

M_w	Energy GJ($\times 10^9$J)	GWh($\times 10^9$Wh)	SEP $\times 3.6 \times 10^{12}$V
4.0	63.095	0.0175	0.0175
4.1	89.125	0.024	0.024
4.2	125.892	0.035	0.035
4.3	177.827	0.049	0.049
4.4	251.188	0.069	0.069
4.5	354.813	0.098	0.098
4.6	501.187	0.139	0.139
4.7	707.945	0.196	0.196
4.8	1000	0.277	0.277
4.9	1412.537	0.392	0.392
5.0	1995.262	0.554	0.554
5.1	2818.382	0.783	0.783
5.2	3981.071	1.106	1.106
5.3	5623.413	1.562	1.562
5.4	7943.282	2.206	2.206
5.5	11220.184	3.116	3.116
5.6	15848.931	4.402	4.402
5.7	22387.211	6.218	6.218
5.8	31622.776	8.784	8.784
5.9	44668.359	12.408	12.408

Table S1. Details of Earthquake energy in joule, watt-hour, and equivalent seismic electrical potential (SEP) (joule/coulomb)

M_w	Energy kJ($\times 10^3$J)	kWh($\times 10^3$Wh)	SEP $\times 3.6 \times 10^{6}$V
0.0	63.095	0.0175	0.0175
0.1	89.125	0.024	0.024
0.2	125.892	0.035	0.035
0.3	177.827	0.049	0.049
0.4	251.188	0.069	0.069
0.5	354.813	0.098	0.098
0.6	501.187	0.139	0.139
0.7	707.945	0.196	0.196
0.8	1000	0.277	0.277
0.9	1412.537	0.392	0.392
1.0	1995.262	0.554	0.554
1.1	2818.382	0.783	0.783
1.2	3981.071	1.106	1.106
1.3	5623.413	1.562	1.562
1.4	7943.282	2.206	2.206
1.5	11220.184	3.116	3.116
1.6	15848.931	4.402	4.402
1.7	22387.211	6.218	6.218
1.8	31622.776	8.784	8.784
1.9	44668.359	12.408	12.408

Table S2. Details of Earthquake energy in Joule, Watt-hour, and equivalent seismic electrical potential (SEP) (joule/coulomb)

M_w	Energy MJ($\times 10^6$J)	MWh($\times 10^6$Wh)	SEP $\times 3.6 \times 10^9$V
2.0	63.095	0.0175	0.0175
2.1	89.125	0.024	0.024
2.2	125.892	0.035	0.035
2.3	177.827	0.049	0.049
2.4	251.188	0.069	0.069
2.5	354.813	0.098	0.098
2.6	501.187	0.139	0.139
2.7	707.945	0.196	0.196
2.8	1000	0.277	0.277
2.9	1412.537	0.392	0.392
3.0	1995.262	0.554	0.554
3.1	2818.382	0.783	0.783
3.2	3981.071	1.106	1.106
3.3	5623.413	1.562	1.562
3.4	7943.282	2.206	2.206
3.5	11220.184	3.116	3.116
3.6	15848.931	4.402	4.402
3.7	22387.211	6.218	6.218
3.8	31622.776	8.784	8.784
3.9	44668.359	12.408	12.408

Table S3. Details of Earthquake energy in joule, watt-hour, and equivalent seismic electrical potential (SEP) (joule/coulomb)

M_w	Energy TJ($\times 10^{12}$J)	TWh($\times 10^{12}$Wh)	SEP $\times 3.6 \times 10^{15}$V
6.0	63.095	0.0175	0.0175
6.1	89.125	0.024	0.024
6.2	125.892	0.035	0.035
6.3	177.827	0.049	0.049
6.4	251.188	0.069	0.069
6.5	354.813	0.098	0.098
6.6	501.187	0.139	0.139
6.7	707.945	0.196	0.196
6.8	1000	0.277	0.277
6.9	1412.537	0.392	0.392
7.0	1995.262	0.554	0.554
7.1	2818.382	0.783	0.783
7.2	3981.071	1.106	1.106
7.3	5623.413	1.562	1.562
7.4	7943.282	2.206	2.206
7.5	11220.184	3.116	3.116
7.6	15848.931	4.402	4.402
7.7	22387.211	6.218	6.218
7.8	31622.776	8.784	8.784
7.9	44668.359	12.408	12.408

Table S4. Details of Earthquake energy in joule, watt-hour, and equivalent seismic electrical potential (SEP) (joule/coulomb)

M_w	Energy PJ($\times 10^{15}$J)	PWh($\times 10^{15}$Wh)	SEP $\times 3.6 \times 10^{18}$V
8.0	63.095	0.0175	0.0175
8.1	89.125	0.024	0.024
8.2	125.892	0.035	0.035
8.3	177.827	0.049	0.049
8.4	251.188	0.069	0.069
8.5	354.813	0.098	0.098
8.6	501.187	0.139	0.139
8.7	707.945	0.196	0.196
8.8	1000	0.277	0.277
8.9	1412.537	0.392	0.392
9.0	1995.262	0.554	0.554
9.1	2818.382	0.783	0.783
9.2	3981.071	1.106	1.106
9.3	5623.413	1.562	1.562
9.4	7943.282	2.206	2.206
9.5	11220.184	3.116	3.116
9.6	15848.931	4.402	4.402
9.7	22387.211	6.218	6.218
9.8	31622.776	8.784	8.784
9.9	44668.359	12.408	12.408
